# Intracranial Pressure during External Ventricular Drainage Weaning Is an Outcome Predictor of Traumatic Brain Injury

**DOI:** 10.1155/2020/8379134

**Published:** 2020-07-09

**Authors:** Jia-cheng Gu, Hong Wu, Xing-zhao Chen, Jun-feng Feng, Guo-yi Gao, Ji-yao Jiang, Qing Mao

**Affiliations:** Department of Neurosurgery, Renji Hospital, School of Medicine, Shanghai Jiao Tong University, Shanghai 200127, China

## Abstract

External ventricular drainage (EVD) is widely used in patients with a traumatic brain injury (TBI). However, the EVD weaning trial protocol varies and insufficient studies focus on the intracranial pressure (ICP) during the weaning trial. We aimed to establish the relationship between ICP during an EVD weaning trial and the outcomes of TBI. We enrolled 37 patients with a TBI with an EVD from July 2018 to September 2019. Among them, 26 were allocated to the favorable outcome group and 11 to the unfavorable outcome group (death, post-traumatic hydrocephalus, persistent vegetative state, and severe disability). Groups were well matched for sex, pupil reactivity, admission Glasgow Coma Scale score, Marshall computed tomography score, modified Fisher score, intraventricular hemorrhage, EVD days, cerebrospinal fluid output before the weaning trial, and the complications. Before and during the weaning trial, we recorded the ICP at 1-hour intervals to calculate the mean ICP, delta ICP, and ICP burden, which was defined as the area under the ICP curve. There were significant between-group differences in the age, surgery types, and intensive care unit days (*p* = 0.045, *p* = 0.028, and *p* = 0.004, respectively). During the weaning trial, 28 (75.7%) patients had an increased ICP. Although there was no significant difference in the mean ICP before and during the weaning trial, the delta ICP was higher in the unfavorable outcome group (*p* = 0.001). Moreover, patients who experienced death and hydrocephalus had a higher ICP burden, which was above 20 mmHg (*p* = 0.016). Receiver operating characteristic analyses demonstrated the predictive ability of these variables (area under the curve [AUC] = 0.818 [*p* = 0.002] for delta ICP and AUC = 0.758 [*p* = 0.038] for ICP burden > 20 mmHg). ICP elevation is common during EVD weaning trials in patients with TBI. ICP-related parameters, including delta ICP and ICP burden, are significant outcome predictors. There is a need for larger prospective studies to further explore the relationship between ICP during EVD weaning trials and TBI outcomes.

## 1. Introduction

External ventricular drainage (EVD) is a common neurosurgical procedure used for subarachnoid hemorrhages, Reye's syndrome, and traumatic brain injury (TBI) [1]. In recent decades, there has been an increased use of EVD catheters coupled with intracranial pressure (ICP) monitoring that allow recording of ICP values and waveforms during continuous cerebrospinal fluid (CSF) drainage [2]. Previous studies have recommended the application of EVD management and ICP monitoring guided by a series of guidelines and consensus [3–5]; however, controversy remains regarding the protocol for EVD removal. Conventionally, an EVD weaning trial precedes EVD removal; however, the weaning protocol varies across institutions with studies placing insufficient emphasis on the ICP during the weaning trial.

Increased ICP has a significant effect on the post-TBI outcomes [6, 7]. Post-traumatic hydrocephalus (PTH), which is among the main TBI complications, as well as the often subsequent ventriculoperitoneal shunt (VPS), has a potential effect on recovery and the quality of life of the patient following recovery. In this prospective study, we hypothesized that ICP during EVD weaning was associated with TBI outcomes. We aimed to assess the changes in the ICP within 24 hours before and during EVD weaning to determine whether our hypothesis was correct.

## 2. Patients and Methods

### 2.1. Patient Selection

This study was reviewed and approved by the Institutional Review Board of Renji Hospital. We enrolled patients with an acute closed TBI who had undergone EVD between July 2018 and September 2019 with or without undergoing other surgery types. The EVD decision was at the discretion of the attending surgeon based on the 4th Edition of the Brain Trauma Foundation's guidelines. All EVD placements were performed in an operating room. Each EVD catheter was coupled with an ICP sensor (Codman, Raynham, Massachusetts) that transmitted ICP signals to the monitor. We excluded patients with intracranial infections, CSF leakage, and severe trauma to other organs, as well as patients who died before the weaning trial or had undergone withdrawal of care during hospitalization.

### 2.2. EVD Weaning Trial Management

After EVD placement, the EVD system was set at 10–20 mmHg with a 100–250 ml daily drainage and routine performance of computer tomography (CT) scans. The starting time of the EVD weaning trial depended on the patient's clinical state, CT scan results, and the physician's judgment. In this medical institute, all physicians abided by the following evaluation principles. If there were no adverse clinical changes (headache, vomiting, and deterioration of consciousness level), no ventricular enlargement or new intracranial hematoma in CT scan, and no progressive increasing ICP, the weaning trial would be performed. To avoid catheter-related infections, the weaning trial began no later than 12–14 days after EVD placement. We employed a rapid-type weaning trial where the EVD was immediately clamped without system height adjustment. In the case of the patient's ICP exceeding 25 mmHg for >5 minutes or the patient's neurological condition deteriorating with a CT scan demonstrating hydrocephalus, the drain was reopened until the ICP dropped below 25 mmHg.

### 2.3. Data Collection

We obtained information on the demographic characteristics (age and sex), pupil reactivity, admission Glasgow Coma Scale (GCS) score, Marshall CT score, modified Fisher score, intraventricular hemorrhage (IVH), surgical treatment types, the length of EVD placement, the length of intensive care unit (ICU) stay, the CSF output before the weaning trial, and the complications (seizure, subdural effusion, deep vein thrombosis, and lung infection). We determined the Marshall CT score, modified Fisher score, and IVH based on the latest preoperative CT scans. We defined the CSF output as the drainage during the last 24 hours before the weaning trial. Regarding the ICP-related parameters, we recorded the ICP value at 1-hour intervals for 24 hours before and during the weaning trial. Each value was recorded under a closed EVD. We used these raw parameters to calculate the mean ICP before and during the weaning trial. The delta ICP was calculated with the following formula: (mean ICP during the weaning trial − mean ICP before the weaning trial)/mean ICP before the weaning trial. Referring to an earlier clinical study on EVD management of TBI patients [8], we defined the ICP burden during the weaning trial as the area under the ICP curve, which was classified as the overall area and the area above the 20 mmHg threshold.

### 2.4. Outcome and Follow-Up

Based on clinical outcomes, we divided the patients into two groups, namely, the favorable and unfavorable outcome groups. The unfavorable outcome group comprised of patients who died and developed PTH and those in a persistent vegetative state or with severe disability. The follow-up was 3 months, with none of the enrolled patients undergoing individual cranioplasty during the follow-up period.

### 2.5. Statistical Analysis

All statistical analyses were performed using a statistical package for social sciences (SPSS) 22.0. Categorical variables were presented as percentages and evaluated using Fisher's two-tailed exact test. Parametric continuous variables were presented as the mean ± standard deviation (SD) and evaluated using Student's two-tailed *t*-test. Nonparametric continuous variables were presented as the median and interquartile range and evaluated using the Mann-Whitney *U* test. We examined factors with significant differences using a receiver operative curve (ROC) to examine their predictive power for the outcome. Statistical significance was set at *p* < 0.05.

## 3. Results

### 3.1. Patient Characteristics

We enrolled 37 patients who completed the 3-month follow-up. Among them, 11 were allocated to the unfavorable outcome group (3 deaths, 4 with PTH requiring a VPS, and 4 in a persistent vegetative state or with severe disability) and 26 to the favorable outcome group.  Table 1 presents the patient characteristics. There were no significant between-group differences in the sex, pupil reactivity, admission GCS score, Marshall CT score, modified Fisher score, IVH, EVD days, CSF output before the weaning trial, and complications. Patients in the unfavorable outcome group were older than those in the favorable outcome group (68 ± 12 vs. 60 ± 11, *p* = 0.045). Twenty (76.9%) patients in the favorable outcome group underwent decompressive craniectomy (DC) with EVD placement, which was significantly higher than the number of patients in the unfavorable outcome group who underwent this procedure (4 [36.4%], *p* = 0.028). Moreover, patients in the unfavorable outcome group had longer ICU stays than those in the favorable outcome group (19 ± 7 vs. 13 ± 5, *p* = 0.004). There were numerically, but not significantly, more patients in the unfavorable outcome group with IVH (6 [54.5%] vs. 5 [19.2%], *p* = 0.051), which might have been limited by the sample size.

### 3.2. Comparison of the Mean ICP

**Figure 1:**
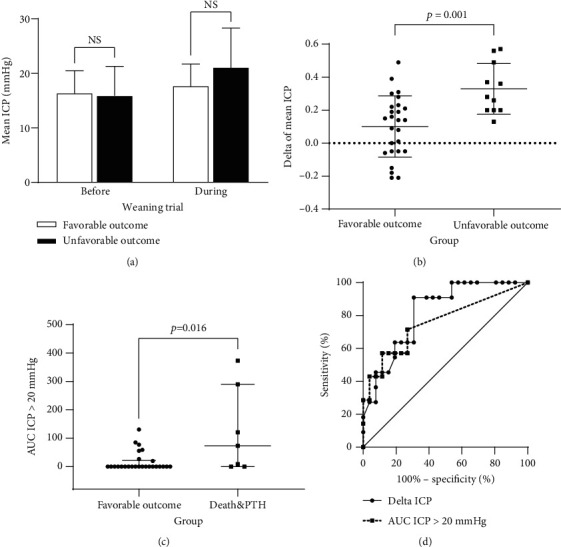
Comparison of the ICP: (a) there is no significant difference in the mean ICP before or during the weaning trial (*p* = 0.780 and *p* = 0.078, *t*-test); (b) the delta ICP is greater in the unfavorable outcome group (*p* = 0.001, *t*-test); (c) AUC for ICP > 20 mmHg is significantly different between the favorable outcome group and patients who died and developed PTH (*p* = 0.016, Mann-Whitney *U* test); (d) ROC analyses showing that the delta ICP has an AUC = 0.818 and *p* = 0.002, while the AUC for ICP > 20 mmHg has an AUC = 0.758 and *p* = 0.038.

As shown in Figure 1(a), patients in both groups had a safe mean ICP before the EVD weaning trial (16.4 ± 4.1, favorable outcome vs. 15.9 ± 5.4, unfavorable outcome). During the weaning trial, 28 (75.7%) patients had an increased ICP, with only 9 (24.3%) patients having an *in situ* or decreased ICP. However, there was an increase in the mean ICP in both groups with no significant between-group differences (17.7 ± 4.0, favorable outcome vs. 21.1 ± 7.2, unfavorable outcome). Moreover, there was no significant difference in the mean ICP before or during the weaning trial (*p* = 0.780 and *p* = 0.078).

### 3.3. Comparison of Delta ICP and ICP Burden

**Figure 2:**
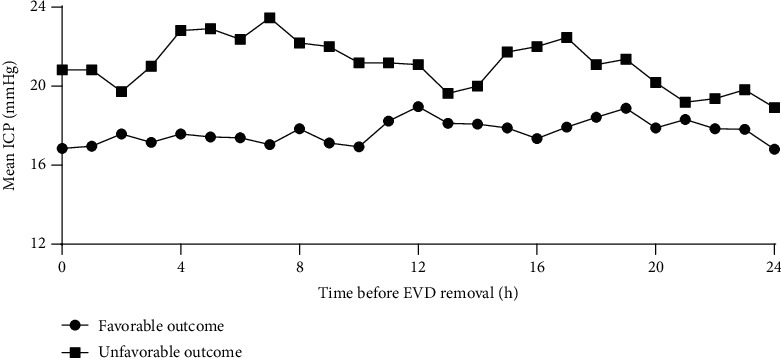
The mean ICP per hour. The mean ICP per hour is higher in the unfavorable outcome group than in the favorable outcome group.

Figure 1(b) shows the delta ICP, which was significantly greater in the unfavorable outcome group (0.33 ± 0.15) than in the favorable outcome group (0.10 ± 0.19, *p* = 0.001). Regarding the ICP burden, there was no significant between-group difference in the overall area under the ICP curve (425.5 ± 97.4, favorable outcome vs. 507.4 ± 173.3, unfavorable outcome; *p* = 0.165). There was no significant between-group difference in the area under curve (AUC) for ICP > 20 mmHg; however, it was significantly different between the favorable outcome group and patients who died or developed PTH (0.0 [0.0, 21.3], favorable outcome vs. 73.0 [0.0, 289.5], death and PTH; *p* = 0.016, Figure 1(c)).  Figure 1(d) presents the ROC analyses of the delta ICP and AUC for ICP > 20 mmHg. The delta ICP had an AUC of 0.818 and a *p* value of 0.002, while the AUC for ICP > 20 mmHg had an AUC of 0.758 and a *p* value of 0.038. As shown in Figure 2, the mean ICP per hour was higher in the unfavorable outcome group than in the favorable outcome group.

## 4. Discussion

In this study, we applied a rapid weaning trial to two groups that were well matched in sex, pupil reactivity, admission GCS score, Marshall CT score, modified Fisher score, IVH, EVD days, CSF output before the weaning trial, and the complications. Consistent with previous findings, we found significant between-group differences in the age, DC surgery, and ICU days. Moreover, the increased age of the patients with TBI is consistent with the characteristics of cities in eastern China. Regarding outcome determination, the PTH proportion was close to the lower limit of the reported range, which could have been limited by our institution's cautious diagnosis and surgical strategy. A PTH diagnosis was only made after observing imaging abnormalities and obvious clinical symptoms. Majority of the patients (28 [75.7%]) presented with an increased ICP during the weaning trial. In both groups, there was an increase in the ICP beyond the 20 mmHg threshold; therefore, it is not enough to simply regard the raw ICP value as a single criterion for weaning trial failure or the outcome. There was no significant difference in the mean ICP before and during the weaning trial, which is consistent with previous findings with other diseases. However, the extended parameters that we assessed demonstrated significant differences and feasible predictive power. The delta ICP was higher in the unfavorable outcome group (*p* = 0.001). Moreover, patients who experienced death and hydrocephalus had a higher ICP burden, which was above 20 mmHg (*p* = 0.016). ROC analyses demonstrated the predictive ability of these variables. There is a need for further studies to clarify this in the context of the pathophysiological mechanism.

EVD was first used in neurosurgery in the 18^th^ century to treat hydrocephalus [9]. Within the last century, there has been an increase in EVD application with a concomitant increasing focus on ICP. Technical improvements have enabled ICP monitoring and EVD management to remain the mainstay treatment options for patients with TBI [3–5]. A weaning trial is conventionally performed prior to EVD removal and allows for evaluation of the self-regulation ability of CSF circulation without an external CSF route. Moreover, it allows for observation of ICP changes based on the Monro-Kellie doctrine [10]. Available literature shows that details regarding the steps and evaluation criteria vary across medical centers and physicians [11, 12]. A randomized trial by Klopfenstein et al. [13] on patients with an aneurysmal subarachnoid hemorrhage (aSAH) compared rapid and gradual weaning protocols. They reported that gradual protocols provided no advantage in preventing long-term shunt placement and prolonged ICU and hospital stays; therefore, rapid weaning is recommended. A recent pooled analysis on 1,171 patients with SAH revealed that gradual weaning decreased the risk of shunt dependency without an additional infection risk; however, this was traded off with longer treatment [14]. There is a need for more studies to determine the optimal weaning protocol and maximal use of physiological parameters during weaning trials.

Despite advances in first aid capability and intensive care management, the reported mortality rates for severe TBI remain high (27%–46%) [15–17]. PTH can develop either in the acute or recovery stage of TBI at a reported rate of 0.7%–51.4% [18], and it often affects the outcome, rehabilitation, and quality of life of patients with TBI. ICP elevation is a significant cause of death and long-term disability resulting from head injuries and other intracranial pathological conditions. A sustained ICP > 20–25 mmHg is considered pathologically significant due to the high correlation between an elevated ICP and mortality [19]. Recent studies have reported that IVH, SAH, age, DC, GCS score, Fisher score, and CSF infection could be risk factors for PTH development [18, 20]. These previous findings represented the observational data for our study.

There have been few studies on whether the physiological parameters during the EVD weaning trial could predict TBI outcomes. There is no available reference evidence for patients with TBI, with most current evidence being obtained from patients with an aSAH or acute hydrocephalus. A recent retrospective study on 94 patients with an aSAH undergoing EVD reported a relationship between the frequency of weaning failure and symptomatic vasospasm with delayed hydrocephalus [21]. A study on 50 patients with an aSAH by Arroyo-Palacios et al. [22] reported that the mean ICP does not affect the weaning outcome; however, the weaning outcome was affected by the characterization of shape differences among ICP pulses. An earlier retrospective study on patients with SAH reported a significant association between weaning failure due to clinical changes and the VPS requirement [23]. However, aSAH and TBI involve different primary damages and pathophysiologies. This indicates that the aforementioned aSAH results cannot be necessarily extrapolated to TBI cases.

Primary damage caused by direct or indirect external forces is the initiating factor of TBI and is often coupled with widespread or perilesional ischemic brain damage [24]. SAH, which is among the primary damage types, can have a direct effect on blood components and cause arachnoid villi impairment. Moreover, there could be a blood clot obstructing the third or fourth ventricles, which could be the mechanism underlying hydrocephalus. At the cellular level, secondary damage involving mitochondrial dysfunction, oxidative stress, cerebral metabolic dysfunction, excitatory amino acids, and endogenous opioid peptides might accelerate apoptosis progression [25, 26]. This subsequently leads to brain damage at the macro level and eventually death. According to the Monro-Kellie doctrine, ICP is mainly affected by cerebrovascular regulation, cerebral perfusion regulation, and regulation of CSF circulation. Increased ICP can lead to a decreased cerebral perfusion pressure, which subsequently leads to significantly decreased cerebral perfusion and flow and ultimately brain infarction and ischemia. Moreover, increased ICP could exacerbate cerebral edema, cerebral hypoxia, hydrocephalus, and brain herniation. PTH is currently thought to be mainly related to decreased CSF absorption due to damaged arachnoid villi, as well as impaired cerebrospinal fluid circulation [27]. For patients undergoing a DC, trauma- and surgery-induced anatomical changes could lead to reduced pulsatile CSF flow and venous outflow, which could be another mechanism underlying PTH. It is evident that an overlap exists between the mechanism of increased ICP and unfavorable TBI outcomes, including death and PTH. This could be a possible explanation for the difference between the favorable outcome group and patients who died and developed PTH with an ICP burden > 20 mmHg in this study. Post-TBI EVD placement allows for both ICP monitoring and diverting of CSF [28]. However, EVD placement impedes the observation of an increased ICP, especially pathophysiological increases [29]. An EVD weaning trial usually takes places within 7–14 days after the first injury. During this period, there is a reduced rebleeding risk, and the peak period for cerebral edema has passed. Moreover, termination of external CSF drainage during this period further reduces the factors influencing ICP. The aforementioned conditions indicate that ICP values are more instructive and could simulate the pathophysiological progress of TBI after EVD removal. The abnormal ICP increase during the weaning trial could be indicative of the body's postinjury deregulation, as well as impeded release of cranial cavity contents after losing the external drainage pathway. In this study, we found that the delta ICP and ICP burden during this period could be reflective of these changes. Before the weaning trial, some patients were clinically stable and their ICP was in a reasonable range. However, they underwent an abnormal elevation in the delta ICP or ICP burden during the weaning trial, and this caused unfavorable outcomes. Therefore, ICP-related parameters during weaning trial could be predictive of the TBI outcome.

There have been few studies on parameters related to the EVD weaning trial in patients with TBI, and a pilot study was required. Although this was a prospective study, it was limited by its small sample size and single-center design. Moreover, a prolonged follow-up period could allow for the evaluation of longer-term and comprehensive outcomes. In this study, we obtained the main indicators from the hourly recorded ICP values, which focused on overall ICP changes and might have neglected sudden increases or short-term fluctuations in the ICP. This could be determined by studying other ICP-related parameters, including the pressure reactivity index, as well as regression of amplitude and pressure. Future studies could assess these indicators to further explore the relationship between ICP during an EVD weaning trial and TBI outcomes.

## 5. Conclusion

ICP elevation is common during the EVD weaning trial in patients with TBI. ICP-related parameters, including delta ICP and ICP burden, are predictive factors of TBI outcomes. There is a need for larger prospective studies to further explore the relationship between ICP during an EVD weaning trial and TBI outcomes.

## Figures and Tables

**Table 1:** Patient characteristics of the study population.

Patient characteristics	Favorable (*n* = 26)	Unfavorable (*n* = 11)	Total (*n* = 37)	*p* value
Sex, *n* (%)				0.244
Male	20 (76.9)	6 (54.5)	26 (70.3)	
Female	6 (23.1)	5 (45.5)	11 (29.7)	
Age (years), mean (SD)	60 (11)	68 (12)	62 (12)	0.045
Pupil reactivity, *n* (%)				0.642
None	1 (3.9)	2 (18.2)	3 (8.1)	
One reactive	5 (19.2)	1 (9.1)	6 (16.2)	
Both reactive	20 (76.9)	8 (72.7)	28 (75.7)	
GCS score, median [IQR]	8.0 [6.0, 11.0]	7.0 [5.0, 8.0]	7.0 [6.0, 9.5]	0.148
Marshall CT score, median [IQR]	6.0 [4.0, 6.0]	4.0 [3.0, 6.0]	6.0 [3.5, 6.0]	0.445
mFisher score, median [IQR]	3.0 [3.0, 3.0]	3.0 [2.0, 3.0]	3.0 [3.0, 3.0]	0.601
IVH, *n* (%)				0.051
Yes	5 (19.2)	6 (54.5)	11 (29.7)	
No	21 (80.8)	5 (45.5)	26 (70.3)	
Surgery type, *n* (%)				0.028
With DC	20 (76.9)	4 (36.4)	24 (64.9)	
Without DC	6 (23.1)	7 (63.6)	13 (35.1)	
EVD days, mean (SD)	7 (2)	9 (3)	7 (2)	0.097
ICU days, mean (SD)	13 (5)	19 (7)	15 (6)	0.004
CSF output (ml), mean (SD)	119 (59)	102 (50)	114 (56)	0.424
Complications, *n* (%)				0.466
Yes	14 (53.8)	8 (72.7)	22 (59.5)	
No	12 (46.2)	3 (27.3)	15 (40.5)	

SD: standard deviation; GCS: Glasgow Coma Scale; IQR: interquartile range; IVH: intraventricular hemorrhage; DC: decompressive craniectomy; EVD: external ventricular drainage; ICU: intensive care unit; CSF: cerebrospinal fluid.

## Data Availability

The datasets generated and/or analyzed during the current study are available from the corresponding author on reasonable request.

## References

[1] Srinivasan V. M., O'Neill B. R., Jho D., Whiting D. M., Oh M. Y. (2014). The history of external ventricular drainage. *Journal of Neurosurgery*.

[2] Chau C. Y. C., Craven C. L., Rubiano A. M. (2019). The evolution of the role of external ventricular drainage in traumatic brain injury. *Journal of Clinical Medicine*.

[3] Carney N., Totten A. M., O'Reilly C. (2017). Guidelines for the management of severe traumatic brain injury, fourth edition. *Neurosurgery*.

[4] Andrews P. J. D., Citerio G., Longhi L. (2008). NICEM consensus on neurological monitoring in acute neurological disease. *Intensive Care Medicine*.

[5] Fried H. I., Nathan B. R., Rowe A. S. (2016). The insertion and management of external ventricular drains: an evidence-based consensus statement : a statement for healthcare professionals from the neurocritical care society. *Neurocritical Care*.

[6] Farahvar A., Gerber L. M., Chiu Y. L. (2011). Response to intracranial hypertension treatment as a predictor of death in patients with severe traumatic brain injury. *Journal of Neurosurgery*.

[7] Vik A., Nag T., Fredriksli O. A. (2008). Relationship of "dose" of intracranial hypertension to outcome in severe traumatic brain injury. *Journal of Neurosurgery*.

[8] Nwachuku E. L., Puccio A. M., Fetzick A. (2014). Intermittent versus continuous cerebrospinal fluid drainage management in adult severe traumatic brain injury: assessment of intracranial pressure burden. *Neurocritical Care*.

[9] Kompanje E. J. O., Delwel E. J. (2003). The first description of a device for repeated external ventricular drainage in the treatment of congenital hydrocephalus, invented in 1744 by Claude-Nicolas Le Cat. *Pediatric Neurosurgery*.

[10] Andrews P. J., Citerio G. (2004). Intracranial pressure. Part one: historical overview and basic concepts. *Intensive Care Medicine*.

[11] Chung D. Y., Leslie-Mazwi T. M., Patel A. B., Rordorf G. A. (2017). Management of external ventricular drains after subarachnoid hemorrhage: a multi-institutional survey. *Neurocritical Care*.

[12] Rao S. S., Chung D. Y., Wolcott Z. (2020). Intermittent CSF drainage and rapid EVD weaning approach after subarachnoid hemorrhage: association with fewer VP shunts and shorter length of stay. *Journal of Neurosurgery*.

[13] Klopfenstein J. D., Kim L. J., Feiz-Erfan I. (2004). Comparison of rapid and gradual weaning from external ventricular drainage in patients with aneurysmal subarachnoid hemorrhage: a prospective randomized trial. *Journal of Neurosurgery*.

[14] Jabbarli R., Pierscianek D., RÖlz R. (2018). Gradual external ventricular drainage weaning reduces the risk of shunt dependency after aneurysmal subarachnoid hemorrhage: a pooled analysis. *Operative Neurosurgery*.

[15] Jiang J.-Y., Gao G. Y., Feng J. F. (2019). Traumatic brain injury in China. *The Lancet Neurology*.

[16] Rosenfeld J. V., Maas A. I., Bragge P., Morganti-Kossmann M. C., Manley G. T., Gruen R. L. (2012). Early management of severe traumatic brain injury. *The Lancet*.

[17] Andriessen T. M. J. C., Horn J., Franschman G. (2011). Epidemiology, severity classification, and outcome of moderate and severe traumatic brain injury: a prospective multicenter study. *Journal of Neurotrauma*.

[18] Schultz B. A., Bellamkonda E. (2017). Management of medical complications during the rehabilitation of moderate-severe traumatic brain injury. *Physical Medicine and Rehabilitation Clinics of North America*.

[19] Balestreri M., Czosnyka M., Hutchinson P. (2006). Impact of intracranial pressure and cerebral perfusion pressure on severe disability and mortality after head injury. *Neurocritical Care*.

[20] de Bonis P., Pompucci A., Mangiola A., Rigante L., Anile C. (2010). Post-traumatic hydrocephalus after decompressive craniectomy: an underestimated risk factor. *Journal of Neurotrauma*.

[21] Akinduro O. O., Vivas-Buitrago T. G., Haranhalli N. (2020). Predictors of ventriculoperitoneal shunting following subarachnoid hemorrhage treated with external ventricular drainage. *Neurocritical Care*.

[22] Arroyo-Palacios J., Rudz M., Fidler R. (2016). Characterization of shape differences among ICP pulses predicts outcome of external ventricular drainage weaning trial. *Neurocritical Care*.

[23] Lewis A., Taylor Kimberly W. (2014). Prediction of ventriculoperitoneal shunt placement based on type of failure during external ventricular drain wean. *Clinical Neurology and Neurosurgery*.

[24] Maas A. I. R., Stocchetti N., Bullock R. (2008). Moderate and severe traumatic brain injury in adults. *The Lancet Neurology*.

[25] Dixon K. J. (2017). Pathophysiology of traumatic brain injury. *Physical Medicine and Rehabilitation Clinics of North America*.

[26] McGinn M. J., Povlishock J. T. (2016). Pathophysiology of traumatic brain injury. *Neurosurgery Clinics of North America*.

[27] Kaur P., Sharma S. (2018). Recent advances in pathophysiology of traumatic brain injury. *Current Neuropharmacology*.

[28] Timofeev I., Dahyot-Fizelier C., Keong N. (2008). Ventriculostomy for control of raised ICP in acute traumatic brain injury. *Acta Neurochirurgica Supplement*.

[29] Exo J., Kochanek P. M., Adelson P. D. (2011). Intracranial pressure-monitoring systems in children with traumatic brain injury: combining therapeutic and diagnostic tools. *Pediatric Critical Care Medicine*.

